# Editorial

**Published:** 1841-12-04

**Authors:** 


### THE MEDICAL EXAMINER.

### PHILADELPHIA, DEC. 4, 1841.

We observe, in the Boston Medical and Surgical Journal for Nov. 24th, that the subcutaneous carving of muscles for lateral curvatures of the spine, is practised at the Orthopedic Infirmary of Boston,—*and are extremely sorry to hear it.*

---

Our highly respected contemporary of the New York Medical Gazette, is hard upon us for the appropriating five columns in our 45th number to "Animal Magnetism;" and, considering us as taking "a middle ground" upon the subject, very pertinently asks whether, in "a periodical which busy doctors are expected to read, *the play is worth the candle!*" *In justice to us*, the editor states, (on our authority, we presume,) "that Professor Wood, of the University, in his late introductory, goes so far in favour of *the new science*, (?) as to consider 'these observations as legitimate subjects for philosophical research,'" &c. It would be scant justice *in us* towards Dr. Wood to charge him with applying the word *science* to any of the vague reveries which have been founded, heretofore, upon a few very curious, but still in some degree isolated facts, having no obvious connection with magnetism, though unwarrantably made to bear the name. We have not even done so *ourselves*. Let us assure our friend of the Gazette, that he will never detect us in shielding our position under the cloak of *authority*, under any circumstances; and we beg to be solely responsible for our philosophical sins. Much that we believe to be true, would undoubtedly be pronounced "humbug" by Dr. Wood, as well as by the editor of the Gazette, were the statement of our opinions suddenly and nakedly advanced. Yet we trust in heaven, *and "our goose-quill grey,"* to make good those opinions to the conviction of our cotemporary himself, when opportunity offers, (we know he is philosophical,) and, also, to do it without the necessity of inditing any thing which, even should our argument prove incorrect, will tax, to no practical benefit, the time of the busiest doctor in "this working day world of ours." When those opinions have been made public through the press, which is not yet the case, none will feel happier than we, to have them *proved*—as almost every thing hitherto based upon the same foundation *has been proved*—a humbug! *We* will venture, then, "always with deference to the editor," to esteem his conclusion *somewhat hasty*. R. C.

---

From what we witnessed in Boston and other cities of Massachusetts, during the intense cold of last winter, we are convinced that the lives of many persons might be saved, in our own more temperate but more variable climate, by the use of the *respirators*. The Boston Journal of the 27th ult. mentions that our friend Dr. Bowditch has a supply of these instruments at various and moderate prices. We cannot quote the prices, for the advertisement of Dr. B. has never met our eye; but to those who are compelled to undergo great exposures while in delicate health, any reference to places where these instruments can be procured of dependable quality, is valuable.

